# Evaluation of postoperative complications of hypospadias using high-frequency ultrasound imaging

**DOI:** 10.1186/s12894-024-01491-y

**Published:** 2024-06-11

**Authors:** Jun Zhuang, Xueshang Su, Ying Jia, Qiaoyuan Zheng, Qingqian Wei, Ziming Zhang, Jintian Hu, Li Yuan, Hongli Chai

**Affiliations:** 1 Department of Ear Reconstruction, Plastic Surgery Hospital, Chinese Academy of Medical Sciences and Peking Union Medical College, Beijing, China; 2 Department of Cosmetic Injection Center, Plastic Surgery Hospital, Chinese Academy of Medical Sciences and Peking Union Medical College, Beijing, China; 3 Ultrasonography Department, Plastic Surgery Hospital, Chinese Academy of Medical Sciences and Peking Union Medical College, Thirty-three Badachu Road, Shijingshan District, Beijing, P.R. China; 4 College of Clinical Medicine, Fudan University, No. 130 Dong’an Road, Xuhui District, Shanghai, 200433 People’s Republic of China

**Keywords:** Complications, Diagnosis, Hypospadias, Ultrasonic

## Abstract

**Purpose:**

Various complications following hypospadias surgery present distinct manifestations when examined with ultrasound. Utilizing high-frequency ultrasound, clinicians can promptly identify these complications and initiate appropriate treatment. The aim of this study is to catalogue the ultrasonographic presentations of various postoperative complications following hypospadias surgery, thereby providing a reference for ultrasonographic diagnosis.

**Methods:**

Ultrasonic images of post-hypospadias surgery from October 1, 2015, to June 30, 2023, recorded at the Plastic Surgery Hospital of the Chinese Academy of Medical Sciences, serve as the basis for this investigation. Drawing on patient clinical diagnoses, this study compiles and selects representative ultrasound images of diverse complications.

**Results:**

The study encompassed a total of 121 subjects; 26 demonstrated urethral stricture on ultrasonic images, two presented local urethral dilation, six showed intraurethral hair-like structures, 17 revealed intraurethral septum, two exhibited intraurethral fold, one had urethral calculus, one displayed urethral calcification, 12 indicated intraurethral urine accumulation, and two showed urethral diverticulum.

**Conclusion:**

Ultrasound examination is helpful for postoperative diagnosis following hypospadias, detecting complications such as urethral stricture, urethral hair growth, and urethral diverticulum, which can help doctors choose appropriate clinical treatment strategies.

## Introduction

Among every 1000 newborns, 0.2 to 4.1 are diagnosed with hypospadias, a prevalent congenital urinary system disorder characterized by the abnormal positioning of the urethral opening on the penis, scrotum, or perineum [1]. Fistulae, diverticula, urethral strictures, and orifice strictures commonly emerge as complications following hypospadias surgery [2–6]. Standard diagnostic techniques include clinical manifestation observation, urethrography, cystoscopy, ultrasonography, computed tomography (CT), and magnetic resonance imaging (MRI) [7–9]. Ultrasonography, due to its noninvasive and convenient nature, facilitates real-time visualization of human tissues. Previous studies have employed ultrasound to evaluate postoperative complications of hypospadias, albeit with a relatively small subject pool [10–12]. This study aims to incorporate a larger patient population and uncover additional ultrasonographic manifestations of postoperative complications associated with hypospadias.

## Methods

The process of urethral ultrasound measurement is primarily bifurcated into two steps. The initial step entails the direct application of an ultrasound probe for the examination of the patient’s urethra. The subsequent step involves the injection of normal saline into the patient’s urethra, followed by the employment of an ultrasound probe for further examination. The study included patients who underwent urethral ultrasound examinations at the Plastic Surgery Hospital of the Chinese Academy of Medical Sciences between October 1, 2015, and June 30, 2023. Exclusion criteria encompassed patients who had not undergone hypospadias surgery and those who were unable to cooperate with the urethral saline injection procedure. All ultrasound images were assessed by a single experienced ultrasonographer. Ultrasound images were captured utilizing a Logic E9 (GE) system operating at a frequency of 15 MHz. This study was approved by the Ethics Committee of the Plastic Surgery Hospital of the Chinese Academy of Medical Sciences and Peking Union Medical College. Ethic Files specific ID numbers: 2024(46). All participants, or their guardians, were informed about and consented to this study.

## Results

**Fig. 1:**
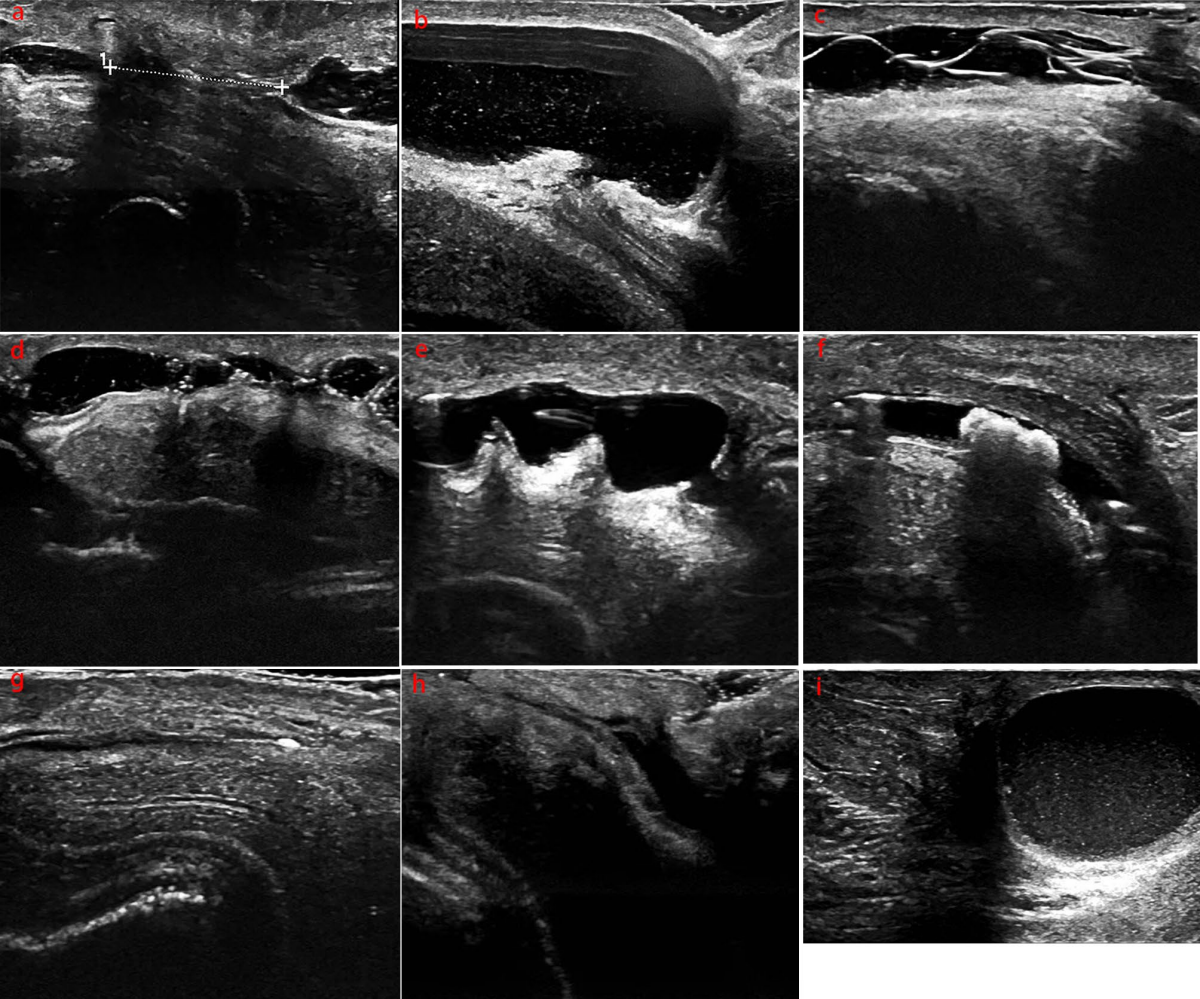
**a**: urethral stricture; **b**: local urethral dilation; **c**:intraurethral hair like structure; **d**: intraurethral septum; **e**: intraurethral fold; **f**:urethral calculus; **g**: urethral calcification; **h**: intraurethral urine accumulation; **i**:urethral diverticulum

**Fig. 2:**
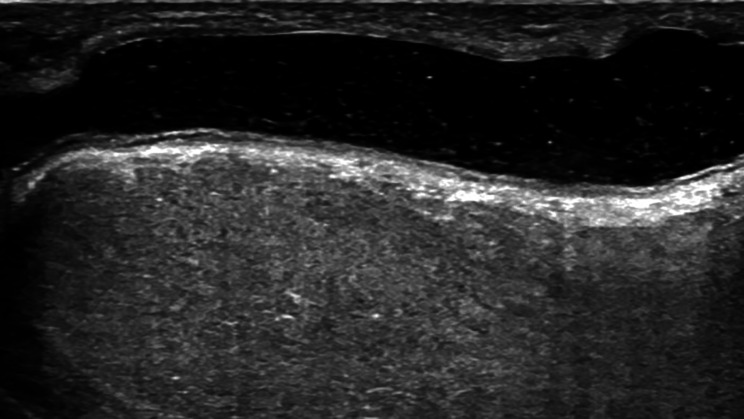
An ultrasound image of a patient with no symptoms after hypospadias surgery

This study included a total of 121 subjects. Subjects ranged in age from 2 to 62 years, with a mean age of 18.06 ± 11.17 years. Ultrasonic findings led to the diagnosis of 26 patients with urethral stricture (Fig. 1a), 2 with local urethral dilation (Fig. 1b), 6 with intraurethral hair-like structures (Fig. 1c), 17 with intraurethral septum (Fig. 1d), 2 with intraurethral fold (Fig. 1e), 1 with urethral calculus (Fig. 1f), 1 with urethral calcification (Fig. 1g), 12 with intraurethral urine accumulation (Fig. 1h), and 2 with urethral diverticulum (Fig. 1i). Additionally, an ultrasound image of a patient exhibiting no symptoms post-hypospadias surgery is also provided (Fig. 2). Detailed ultrasound descriptions and proportions of these complications are presented in Table 1.

**Table 1:** Detailed ultrasound description and proportion of postoperative complications

Diagnosis	Ultrasonography	Proportion
Urethral stricture	Under normal circumstances, part of the urethra is visible, but after injecting liquid substances, poor dilation may occur in the local urethra.	26/121
Local urethral dilation	Local dilation of the urethra is visible, sound transparent is acceptable, the anterior wall is not smooth.	2/121
Intraurethral hair	Under normal circumstances, part of the urethra is visible, with no obvious residual urine echo in both bulbar urethra and penile urethra before injecting liquid substances. After the injection, the entire process of urethra is slightly dilated with no significant enlargemt. Hair like echoes can be seen inside.	6/121
Intraurethral septum	Under normal circumstances, part of the urethra is visible, with no obvious residual urine echo in both bulbar urethra and penile urethra before injecting liquid substances. After the injection, the entire process of urethra is slightly dilated with no significant enlargemt. A septum like structure can be seen inside.	17/121
Intraurethral fold	Under normal circumstances, part of the urethra is visible, with no obvious residual urine echo in both bulbar urethra and penile urethra before injecting liquid substances. After the injection, the entire process of urethra is slightly dilated with no significant enlargemt. Several fold like structure can be seen inside. No obvious residual urine can be seen in the bladder.	2/121
Urethral calculus	Under normal circumstances, part of the urethra is visible, with a strong intraurethral echo accompanied by a ultrasonic shadow. No obvious residual urine echo can be seen in both bulbar urethra and penile urethra before injecting liquid substances. After the injection, the entire process of urethra is slightly dilated with no significant enlargemt. The strong echo appears movable.	1/121
Urethral calcification	Under normal circumstances, part of the urethra is visible, with no obvious residual urine echo in penile urethra before injecting liquid substances, but strong echoes can be seen inside the posterior urethra. After the injection, the entire process of urethra is slightly dilated with no significant enlargemt.	1/121
Intraurethral urine accumulation	Before urination, local liquid echoes can be seen inside the urethra. After urination, intraurethral liquid echoes significantly increase in comparison with before urination.	2/121
Urethral diverticulum	A cystic structure can be seen in the ventral side of urethra between penis and scrotum, which is connected with the urethra.	2/121

## Discussion

Hypospadias is a congenital condition that refers to the abnormal positioning of the urethral opening in the male reproductive organ. Surgery for hypospadias serves as the primary approach for managing this condition. Despite surgery being the primary treatment, postoperative complications such as urethral stricture, urethral fistula, and infection can still occur [13]. Postoperative ultrasound examination is crucial in the management of hypospadias as it provides essential information to help physicians evaluate the effectiveness of the surgical repair, detect complications, and follow up on the treatment outcome. Ultrasonography can reveal urethral conditions in real time [14]. The injection of saline into the urethra not only discloses the urethra’s normal condition but also simulates urination, thereby underscoring the importance of ultrasonography in assessing urethral conditions. The discharge of urine is an important observation index after urethral reconstruction surgery [15]. Via ultrasound examination, physicians can observe the flow of urine in the urethra and evaluate the smoothness of urine discharge. Should difficulties in urination or abnormal urine reflux be detected, physicians can implement timely interventions to ensure normal urine discharge. Postoperative urethral stricture, a common complication of hypospadias surgery, often manifests as difficulty with local dilation or dilation challenges associated with a local bulge in the anterior region. Patients might experience difficulties in urinating. Moreover, the accumulation of a substantial volume of urine in the bladder can stimulate the bladder’s nerves, resulting in symptoms such as frequent urination and urgency [16]. Isolated clinical manifestations may not accurately reflect the actual condition of urethral strictures, while ultrasonography, when combined with intraurethral liquid injection, can unveil the specific location and degree of stenosis. Manifestations of urethral diverticula encompass hematuria, urethral infections, urethral calculi, dysuria, and potentially carcinoma. Retrograde urography or MRI can be utilized for the diagnosis of urethral diverticula [17, 18]. Compared to the aforementioned methods, ultrasound, being economical and convenient, and lacking radiation, offers real-time insights into urethral conditions. This assists doctors in estimating the size and location of diverticula, serving as a reference for subsequent treatment steps. Postoperative intraurethral hair growth presents another challenging issue, leading to dysuria and penile pain. Traditionally, intraurethral hair has been treated with surgical resection; however, recent findings suggest lasers can also effectively remove hair from the urethra [19, 20]. Ultrasonography distinctly reveals intraurethral hair-like structures, aiding doctors in making initial assessments and guiding further treatment. Furthermore, given the necessity for long-term follow-up post-hair removal, ultrasonography offers a straightforward examination method. Persistent dysuria, residual urine, and infections may lead to urethral calcification and calculi formation. Calcification lesions exhibit a strong echo on ultrasound, whereas calculi present a strong echo accompanied by a posterior shadow, attributed to their high tissue density. Furthermore, it is advisable for doctors to instruct patients to void their bladder before undergoing urethral ultrasonography and to measure the residual urine during the procedure, thereby assessing their urinary conditions. The results of urethral ultrasonography should be interpreted in conjunction with patients’ clinical manifestations and medical history, as some may report dysuria or other symptoms even when ultrasonic findings indicate no anomalies. Ultrasound can further assist doctors in evaluating the urethral wall by observing its structure, such as thickness and smoothness, to assess the healing status of the surgical site [21]. A well-healed urethral wall will exhibit a uniform and continuous structure, whereas poor healing or fistulas may result in interrupted or irregular continuity of the urethra, clearly discernible through ultrasound examination. Finally, given that ultrasound does not expose patients to radiation and is convenient to perform and repeat [22], it plays a pivotal role in the follow-up treatment after urethral repair surgery. Through regular ultrasound examinations, doctors can evaluate the durability of treatment effects and promptly identify and manage potential complications.

## Limitation

However, ultrasound also has limitations in evaluating the postoperative condition of urethral reconstruction. Owing to the small size of the urethral structure and limited resolution of ultrasound images, it may not be able to distinguish some deep and subtle structures. For instance, while ultrasound examination can quickly and effectively observe the periurethral structures, its ability to observe the internal structure of the urethra is not as effective as urethroscopy. For patients whose conditions cannot be adequately assessed by ultrasound, additional examinations such as urethroscopy, CR, or MRI may be necessary. However, ultrasound examination also presents distinct advantages. The use of urethral ultrasound is gentler for patients and does not cause damage to the tissues inside the urethra. It can circumvent complications such as bleeding and infection caused by urethroscopy, thereby making it more acceptable for patients, particularly children. Moreover, hypospadias is characterized by three features: a ventral opening of the meatus, chordee (curvature), and an absence of ventral prepuce. The specific preoperative conditions vary among patients. However, as this study included only patients who underwent ultrasound examination post-hypospadias surgery, many were uncertain about the specific subtypes of their hypospadias; thus, these subtypes were not analyzed. Furthermore, our results are also limited due to a lack of non-complicated repair group comparison (in terms of statistical analysis). To include a broader patient cohort and acquire a more extensive collection of complication cases and their ultrasound images, the relationship between the postoperative interval and the occurrence of complications was not analyzed in this study. Moreover, a substantial proportion of patients in this study developed complications or underwent follow-up several years, or even decades, post-surgery, contributing to a higher average age.

## Conclusion

Ultrasound examination is helpful for postoperative diagnosis following hypospadias, detecting complications such as urethral stricture, urethral hair growth, and urethral diverticulum. However, the use of ultrasound for follow-up of hypospadias is not yet necessary, and not all clinical doctors will routinely use it. Moreover, it is highly user dependent, and is not likely to be immediately useful to untrained clinicians. This study is only a descriptive study that demonstrates the value of ultrasound examination, and more quantifiable data is needed to supplement it in the future.

## Data Availability

The datasets used and/or analysed during the current study available from the corresponding author on reasonable request.
